# Dynamic Magnetic Properties of Germole‐ligated Lanthanide Sandwich Complexes

**DOI:** 10.1002/chem.202300567

**Published:** 2023-05-11

**Authors:** Siddhartha De, Arpan Mondal, Ze‐Yu Ruan, Ming‐Liang Tong, Richard A. Layfield

**Affiliations:** ^1^ Department of Chemistry School of Life Sciences University of Sussex Brighton BN1 9QR UK; ^2^ Key Laboratory of Bioinorganic and Synthetic Chemistry of the Ministry of Education School of Chemistry Sun-Yat Sen University Guangzhou 510006 P. R. China

**Keywords:** germole, hysteresis, lanthanides, sandwich complexes, single-molecule magnets

## Abstract

The first germole‐ligated single‐molecule magnets are reported, with contrasting properties found for the near‐linear sandwich complexes [(η^8^‐COT)Ln(η^5^‐Cp^Ge^]^−^, where Ln=Dy (**1_Dy_**) or Er (**1_Er_**), COT is cyclo‐octatetraenyl and Cp^Ge^ is [GeC_4_‐2,5‐(SiMe_3_)_2_‐3,4‐Me_2_]^2−^. Whereas **1_Er_** has an energy barrier of 120(1) cm^−1^ in zero applied field and open hysteresis loops up to 10 K, the relaxation in **1_Dy_** is characterized by quantum tunneling within the ground state.

## Graphical abstract

**Single‐molecule magnets**: The first examples of germole‐ligated lanthanide single‐molecule magnets are reported, with markedly different properties found for the dysprosium and erbium versions.

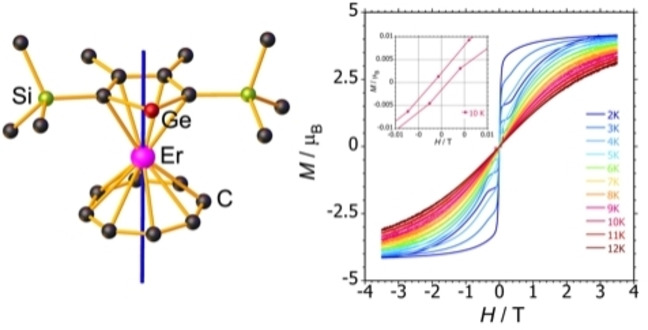

## Introduction

Single‐molecule magnets (SMMs) containing highly anisotropic metal ions have generated considerable interest in recent years owing to their slow magnetic relaxation, magnetic bistability and other valuable properties.^[1]^ Applications of SMMs in prototype molecular spintronic materials have also been reported.^[2]^ In the case of monometallic lanthanide SMMs, single‐ion magnetic anisotropy, coordination symmetry and spin‐phonon interactions have been found to determine the overall SMM performance, in addition to specific features of the various relaxation pathways.^[3]^ Of the different subclasses, lanthanide organometallic sandwich and half‐sandwich SMMs have been known for over a decade.^[4]^ Cyclopentadienyl (Cp) complexes of dysprosium have risen to prominence, with many leading SMMs showing high magnetic blocking temperatures and large effective energy barriers being based on the {(η^5^‐Cp)_2_Dy} structural motif.^[5]^ Cyclo‐octatetraenyl (COT) complexes of erbium have also provided important insight into the ligand field picture in lanthanide SMMs.^[6]^ Other carbon‐based donor ligands, such as cyclobutadienyl,^[7]^ cycloheptatrienyl^[8]^ and cyclononatetraenyl^[9]^ have recently been incorporated into the lanthanide SMM family.

An emerging trend in the development of organometallic SMMs is the replacement of a {CR} unit (R=H, alkyl, aryl, silyl) from a cyclopentadienyl ligand with an isolobal and valence isoelectronic heteroatom.^[10]^ Such replacements allow the basic metallocene structure to be maintained while modifying the axial crystal field experienced by the lanthanide. For example, dysprosium SMMs with dianionic borolide ligands^[11]^ ([C_4_R_4_B]^2−^) and erbium SMMs containing a plumbole ligand^[12]^ ([C_4_R_4_Pb]^2−^) have been described. The borolide‐ligated SMMs possess very high effective barriers and magnetic blocking temperatures. More generally, the coordination chemistry of dianionic metallole ligands ([C_4_R_4_E]^2−^, E=Si−Pb) towards rare earth elements is an emerging area, and we were interested in gaining insight into the impact of a germole ligand of the type [C_4_R_4_Ge]^2−^ on the magnetism and electronic structure of dysprosium and erbium.

## Results and Discussion

**Scheme 1:**
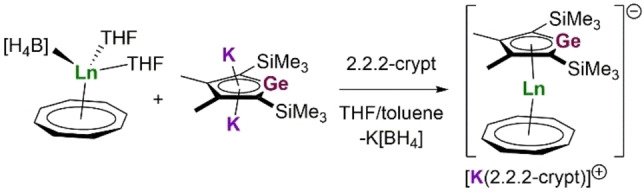
Synthesis of [K(2.2.2‐crypt)][**1_Ln_**] (Ln=Dy, Er).

The target complexes [(η^8^‐COT)Ln(η^5^‐Cp^Ge^)]^−^, where Ln=Dy (**1_Dy_**) or Er (**1_Er_**) and Cp^Ge^=[GeC_4_‐2,5‐(SiMe_3_)_2_‐3,4‐Me_2_]^2−^, were synthesized as salts of [K(2.2.2‐crypt)]^+^ by adding [K_2_Cp^Ge^⋅0.75THF] to a solution of [(η^8^‐COT)Ln(BH_4_)(THF)_2_] in a 1 : 1 mixture of THF and toluene at room temperature, followed by refluxing for 16 h (Scheme 1). Crystals of **1_Dy_** and **1_Er_** were isolated in yields of 61 % and 50 %, respectively.

**Figure 1:**
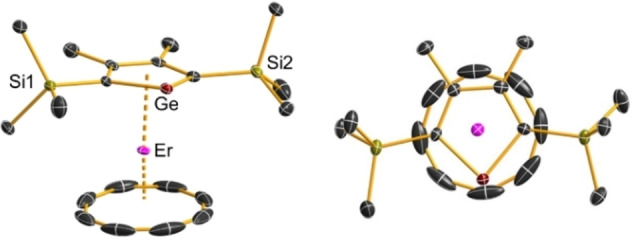
Thermal ellipsoid representation (50 % probability) of the structure of **1_Er_** anion. For clarity, carbon atoms in black are unlabeled and hydrogen atoms are not shown.

The molecular structures of **1_Dy_** and **1_Er_** were determined by X‐ray crystallography and found to be very similar (Tables S1–S3, Figures 1, S3 and S4). Both complexes are near‐linear metallocene‐like sandwiches, with η^8^‐coordination of the COT ligand and skewed η^5^‐coordination of the germole ligand owing to the relatively long Ln⋅⋅⋅Ge distances. In **1_Dy_**, the Dy−C distances to the COT ligand lie in the range 2.546(4)–2.575(5) Å and the Dy−COT distance to the ligand centroid is 1.7956(2) Å. The Dy−C and Dy−Ge distances are 2.620(4)‐2.637(4) Å and 2.9424(5) Å, respectively, and the Dy−Cp^Ge^ distance to the ligand centroid is 2.3063(2) Å. The COT−Dy−Cp^Ge^ angle is 175.135(12)°. Key structural parameters for **1_Er_** are Er−C distances of 2.523(3)–2.560(3) Å to the COT ligand, an Er−COT centroid distance of 1.7610(13) Å, Er−C distances of 2.589(2)–2.602(2) Å to the germole ligand, an Er−Ge distance of 2.9152(3) Å, and an Er−Cp^Ge^ centroid distance of 2.2693(10) Å. The COT−Er−Cp^Ge^ angle is 175.38(5)°. The relatively short distances in **1_Er_** relative to **1_Dy_** reflect the slightly smaller ionic radius of Er^3+^ when compared to Dy^3+^.

Germole complexes of rare earth elements are extremely uncommon. The first examples, which contain either yttrium, lanthanum, or cerium, were reported only recently.^[13]^ In these systems, direct interactions between the germanium‐centred lone pair and the rare earth element result in structural aggregation, typified by the coordination polymer of dimers [{K(THF)(μ:η^8^:η^8^‐COT)La(η^5^‐Cp^Ge^)}_2_]_∞_,^[13b]^ and a perturbation of the axial metallocene crystal field by an equatorial germole ligand. The absence of these interactions from the structures of **1_Dy_** and **1_Er_** mean that these complexes are the first lanthanide germole sandwich complexes to display pseudo‐axial symmetry and to contain metals relevant to single‐molecule magnetism. The magnetic susceptibility properties of both complexes were therefore studied in static (DC) and dynamic (AC) magnetic fields.

In an applied DC field of 1000 Oe, the value of *χ*_M_ *T* (*χ*_M_ is the molar magnetic susceptibility) for **1_Dy_** at 300 K is 13.58 cm^3^ K mol^−1^ and, therefore, very similar to the theoretical value of 14.1 cm^3^ K mol^−1^ for Dy^3+^ with a ^6^H_15/2_ ground term (Figure S5). On lowering the temperature, *χ*_M_ *T* decreases gradually before decreasing more rapidly below 100 K to reach 8.84 cm^3^ K mol^−1^ at 2 K. In the case of **1_Er_**, *χ*_M_ *T* is 11.17 cm^3^ K mol^−1^ at 300 K, consistent with a ^4^I_15/2_ ground term, and follows a similar profile to **1_Dy_** in the region 100–300 K (Figure S6). Below 100 K, the susceptibility decreases in a near‐linear manner down to 4 K, followed by a precipitous drop to reach 7.19 cm^3^ K mol^−1^ at 2 K. The contrasting low‐temperature susceptibility of the two compounds can be interpreted in terms of magnetic blocking and single‐molecule magnetism in **1_Er_** but not in **1_Dy_**. This notion is supported by the observation of bifurcation of the FC‐ZFC (field‐cooled, zero field‐cooled) susceptibility around 5 K for **1_Er_**, whereas no such behavior was observed for **1_Dy_** (Figures S7 and S8).

**Figure 2:**
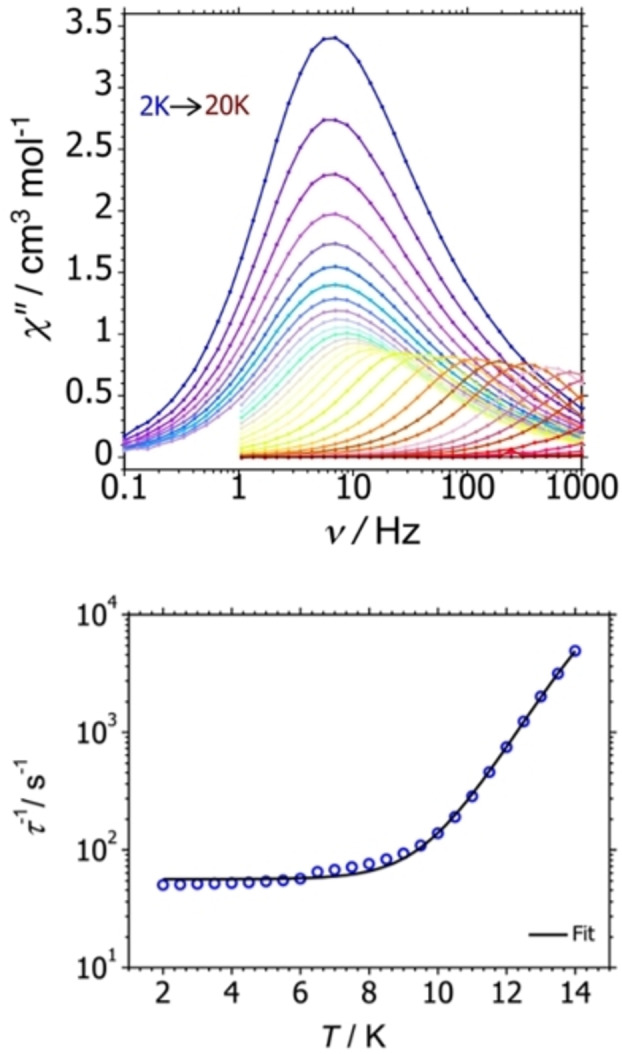
Top: frequency dependence of the out‐of‐phase magnetic susceptibility (*χ*′′) for **1_Er_** in zero DC field at *ν*=1–1000 Hz and temperatures of 2–20 K. Bottom: Plot of $\tau^{-1}$ vs. temperature for **1_Er_**, where the solid line is a fit to the data using the parameters stated in the text.

Confirmation of the SMM properties of **1_Er_** was obtained from AC susceptibility measurements in zero DC field (Figures S9–S13, Table S4). Maxima were observed in the frequency‐dependence of the imaginary component of the AC susceptibility, i. e., *χ*′′(*ν*) (Figure 2). The position of the peak maximum is essentially temperature independent in the range 2–7 K, before showing a strong temperature up to 14 K, at which point the upper frequency limit of 1000 Hz for the susceptometer is reached. The plot of the inverse relaxation time ($\tau^{-1}$ ) against temperature in the region 2–14 K (Figure 2) shows a linear relationship at higher temperatures, a temperature‐independent relationship at lower temperatures, and curvature at intermediate temperatures. These data were therefore fitted using Orbach, Raman and QTM (quantum tunnelling of the magnetization) terms, i. e. $\tau^{-1}=\;\tau_{0}^{-1}e^{-U_{eff}/k_{B}T}+CT^{n}+\tau_{QTM}^{-1}$ , in which $\tau_{0}$ is the attempt time, *U*_eff_ is the effective energy barrier, *C* and *n* are the Raman coefficient and exponent, respectively, and $\tau_{QTM}^{-1}$ is the rate of QTM. The fit parameters obtained from this analysis are $\tau_{0}$ =10^−9.02(4)^ s, *U*_eff_=120(1) cm^−1^, *C*=10^−1.6(4)^ s^−1^ K^−*n*^, *n*=3.4(4) and $\tau_{QTM}$ =10^−1.7(1)^ s. In contrast, no maxima were visible in the *χ*′′(*ν*) data for **1_Dy_** within the frequency window accessible to our susceptometer in zero DC field, hence SMM behaviour could not be determined (Figures S16–S19). This observation is broadly consistent with previously reported COT complexes of Dy^3+^.[^6f^, ^9^]

Complex **1_Er_** is a new member of the family of erbium SMMs with the general formula [(η^8^‐COT)Er(η^5^‐Cp^E^], where Cp^E^ is a cyclopentadienyl, phospholyl or plumbole ligand.[^10c^, ^12^, ^14^] The characterization of **1_Er_** result provides a new opportunity to interpret the SMM properties with a qualitative model that considers interactions between the prolate‐shaped 4 f electron density of Er^3+^ and the crystal field. Here, we use [(η^8^‐COT)Er(η^5^‐Cp^ttt^)] (**2**, Cp^ttt^=1,2,4‐C_5_^*t*^Bu_3_H_2_) as the benchmark since this SMM shows a single thermally activated Orbach process with *U*_eff_=228.26 cm^−1^.^[14a]^

The structure of **2** features an Er−COT centroid distance of 1.718 Å, which is 0.04 Å shorter than in **1_Er_**, and an Er−Cp^ttt^ distance of 2.310 Å, which is 0.04 Å longer than in **1_Er_**. The COT−Er−Cp^Ge^ angles in the two complexes are essentially the same. These distances can be rationalized in terms of a stronger interaction between the dianionic and relatively charge dense germole ligand and erbium, which results in the COT ligand in **1_Er_** being pushed away from the metal. Since the magnetic anisotropy and crystal field splitting of Er^3+^ are enhanced in equatorial crystal fields, the scenario for **1_Er_** means that the axial crystal field provided by the Cp‐like ligand is stronger than in **2** and the equatorial crystal field provided by the COT ligand is weaker. Hence, the effective barrier for **1_Er_** should be smaller than in **2** and the rate of QTM should be faster, as we observe. We note that the energy barrier of 120(1) cm^−1^ for **1_Er_** is much larger than the barrier of 42 cm^−1^ determined for the plumbole‐ligated SMM [{η^5^‐PbC_4_Ph_2_(SiMe_2_^t^Bu)_2_}Er(η^8^‐COT^Tips^)]^−^ (Tips=triisopropylsilyl), which could indicate that different heteroatoms can be used to fine‐tune the properties. However, the use of bulkier substituents in the erbium−plumbole SMM is also likely to play a role in weakening the crystal field by pushing both ligands away from the lanthanide.^[12]^

**Figure 3:**
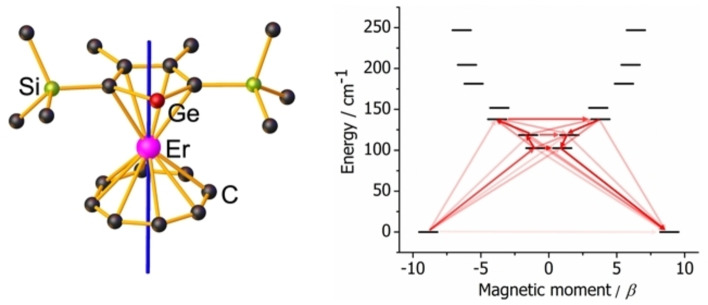
Left: principal magnetic axis of the ground Kramers doublet in 1Er (pink=Er, red=Ge, green=Si, back=C, H atoms not shown). Right: Calculated relaxation barrier for 1Er. Stronger red arrows indicate larger absolute value of the transition magnetic moment matrix elements. Transitions involving higher‐energy states are omitted for clarity.

Detailed analysis of the electronic structure of **1_Er_** and **1_Dy_** was undertaken using multireference ab initio calculations. For both complexes, the calculated magnetic susceptibility and isothermal field‐dependence of the magnetization closely match the experimental data (Figures S5 and S6). The energies of the eight Kramers doublets (KDs) within the respective ^4^I_15/2_ and ^6^H_15/2_ ground multiplets of Er^3+^ and Dy^3+^ are shown in Table S5. The principal magnetic axis in the ground KD of **1_Er_** roughly coincides with the COT−Er−Cp^Ge^ axis, whereas in **1_Dy_** it is skewed towards the edges of the two ring systems (Figure 3).

The ground KD for **1_Er_** has strong axial character, with *g_x_* =0.003, *g_y_* =0.004 and *g_z_* =17.74, and is essentially characterized solely by |*M_J_* |=15/2 (Table S6). In contrast, the first‐excited KD at 103 cm^−1^ is non‐axial, with *g_x_* =11.61, *g_y_* =6.92 and *g_z_* =1.15, with significant mixing of *M_J_* states. Consequently, the most probable thermally activated relaxation pathway for 1_Er_ is via the first‐excited KD (Figure 3, Table S8), which is in reasonable agreement with the experimental *U*_eff_ value. The appreciable wavefunction mixing in the first‐excited and higher‐lying KDs in 1_Er_ is reflected in the calculated axial $B_{2}^{0}$ and non‐axial $B_{2}^{2}$ crystal field parameters, which are of comparable magnitude, whereas the higher‐order axial parameters $B_{k}^{0}$ (*k*=4, 6) are significantly smaller (Table S10). A similar analysis applied to **1_Dy_** reveals why this complex does not give rise to SMM behaviour. Appreciable admixture of different *M_J_* states occurs in the ground KD, which also features appreciable transverse components to the g‐tensors, i. e., *g_x_* =0.041, *g_y_* =0.067 and *g_z_* =18.02 (Table S7). Hence, a barrier‐crossing transition within the ground KD (i. e., QTM) is the most probable relaxation pathway (Table S9).

**Figure 4:**
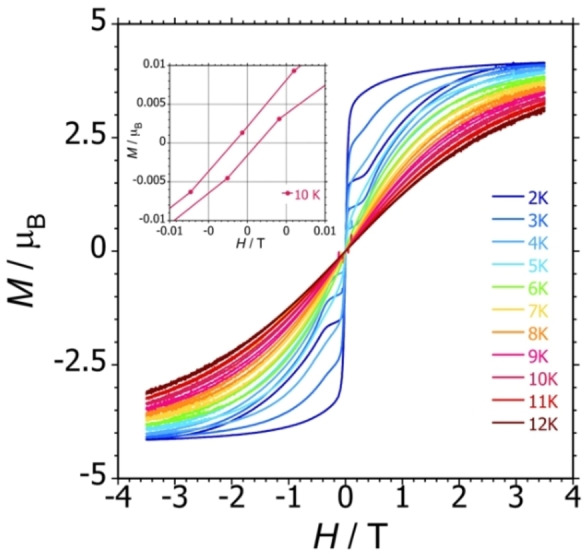
Magnetic hysteresis loops for **1_Er_** with an average field‐sweeping rate of 20 mT s^−1^.

The contrasting AC susceptibility properties of **1_Er_** and **1_Dy_** are also mirrored in the magnetic hysteresis. Using an average field sweep rate of 200 Oe s^−1^ (20 mT s^−1^), S‐shaped hysteresis loops were observed for **1_Er_** in the temperature range 2–10 K (Figure 4). At 2 K, appreciable opening of the loops occurs at fields up to 3 kOe, but with significant constriction of the loops around zero field. In the case of **1_Dy_**, no significant opening of the loops at 2 K was observed (Figure S20). The hysteresis properties of the germole‐ligated complexes are therefore consistent with those of previously reported [(η^8^‐COT)Er(η^5^‐Cp^E^)] SMMs.

## Conclusions

In conclusion, the germole‐ligated erbium and dysprosium sandwich compounds [K(2.2.2‐crypt)][**1_Er_**] and [K(2.2.2‐crypt)][**1_Dy_**] have been synthesized and studied using magnetic property measurements and ab initio calculations. Whereas no SMM behavior originates from **1_Dy_**, an energy barrier of 120(1) cm^−1^ in zero applied field was determined for **1_Er_**, along with open magnetic hysteresis loops up to 10 K. A key finding from our studies of **1_Er_** and **1_Dy_**, which is reinforced through comparison with related SMMs, is that the dianionic germole ligand can indeed enforce a strong axial crystal field that seemingly dominates over the COT ligand and relative to cyclopentadienyl ligands in complexes of the type [(η^8^‐COT)Er(η^5^‐Cp^E^]. Thus, when combined with another ligand that provides an axial crystal field, germole ligands should have potential as the basis of high‐performance terbium or dysprosium SMMs, provided an axial geometry is maintained and provided the germanium‐centred lone‐pair does not engage in dative bonding with nearby lanthanide ions. Current efforts in our laboratory are focused on the synthesis of such materials.

## Supporting Information

Synthesis, tables for crystallography, FTIR spectra, magnetic characterization, and computational data of compound **1_Dy_** and **1_Er_** can be found in the Supporting Information.

Deposition Number(s) 2223352 (for **1**_**Dy**_) and 2223351(for **1**_**Er**_) contain(s) the supplementary crystallographic data for this paper. These data are provided free of charge by the joint Cambridge Crystallographic Data Centre and Fachinformationszentrum Karlsruhe Access Structures service.

Additional references cited within the Supporting Information.[^15^, ^16^, ^17^, ^18^, ^19^, ^20^, ^21^, ^22^, ^23^, ^24^, ^25^, ^26^, ^27^, ^28^] The data that support the findings of this study are openly available at 10.25377/sussex.22555429.

## Conflict of interest

The authors declare no conflict of interest.

1

## Supporting information

As a service to our authors and readers, this journal provides supporting information supplied by the authors. Such materials are peer reviewed and may be re‐organized for online delivery, but are not copy‐edited or typeset. Technical support issues arising from supporting information (other than missing files) should be addressed to the authors.

Supporting Information

## Data Availability

The data that support the findings of this study are openly available in sussex.figshare.com at https://doi.org/10.25377/sussex.22555429, reference number 22555429.
