# Supplementary material for: Dynamic Magnetic Properties of Germole‐ligated Lanthanide Sandwich Complexes
Source: Chemistry. 2023 May 11;29(37):e202300567. doi: 10.1002/chem.202300567 (PMC10947301; doi:10.1002/chem.202300567)
Supplement: Supplementary file 1 — Supporting Information [file CHEM-29-0-s001.pdf]

Chemistry–A European Journal

Supporting Information

Dynamic Magnetic Properties of Germole-ligated Lanthanide Sandwich Complexes

Siddhartha De, Arpan Mondal, Ze-Yu Ruan, Ming-Liang Tong,* and Richard A. Layfield*

General Considerations

All manipulations were carried out under anhydrous and anaerobic conditions using standard Schlenk line techniques and argon-filled glove boxes. Solvents were refluxed over an appropriate drying agent for a minimum of three days (molten potassium for toluene, THF and Na/K alloy for hexane), and then distilled, degassed via a minimum of three freeze-pump-thaw cycles, and stored in ampoules over potassium mirrors (toluene and hexane) and activated 4 Å molecular sieves (THF). The dipotassium salt of the germole (K_2Cp^{Ge}) and [Ln(COT)(BH_4)($THF)_2$] (Ln = Dy, Er) were prepared according to a literature procedure.[15][16] 2.2.2-Cryptand was purchased from Sigma-Aldrich and dried under vacuum at 60 °C for 3 days prior to use. All other chemicals were obtained from commercial sources and used without further purification.

Elemental analyses were carried out at Microanalytisches Labor Pascher, Germany. Attenuated total reflectance Fourier-transform infrared spectroscopy (ATR-FTIR) spectra were collected using a Bruker ALPHA spectrometer equipped with a Platinum ATR module in an argon- filled glovebox.

Synthesis of [K(2.2.2-crypt)][(η^8-COT)Dy(η^5-Cp^{Ge}] ([K(2.2.2-crypt)][$\mathbf{1_{Dy}}$])

THF (3 ml) was added to a mixture of [K_2Cp^{Ge}·0.75THF] (22 mg, 0.05 mmol), [Dy(COT)(BH_4)($THF)_2$] (22 mg, 0.05 mmol) and 2.2.2-cryptand (19 mg, 0.05 mmol) at room temperature. After stirring for 15 minutes, toluene (3 ml) was added and the mixture was heated for 16 hours at 110 °C. The resultant red suspension was allowed to settle for 30 minutes and filtered. The filtrate was evaporated to dryness and extracted with THF. The THF-soluble fraction was layered with hexane at room temperature. Red crystals of [K(2.2.2-crypt)][$\mathbf{1_{Dy}}$] were isolated after five days (30 mg, 61%). Elemental analysis calculated for $C_{38}H_{68}DyGeSi_2KN_2O_6$: C, 46.60; H, 7.00; N, 2.86. Found: C, 46.70; H, 7.10; N, 3.02.

Synthesis of [K(2.2.2-crypt)][(η^8-COT)Er(η^5-Cp^{Ge}] ([K(2.2.2-crypt)][$\mathbf{1_{Er}}$])

Using the method described above and [K_2Cp^{Ge}·0.75THF] (22 mg, 0.05 mmol), [Er(COT)(BH_4)($THF)_2$] (22 mg, 0.05 mmol) and 2.2.2-cryptand (19 mg, 0.05 mmol), orange-red crystals of [K(2.2.2-crypt)][$\mathbf{1_{Er}}$] were isolated (24 mg, 49%). Elemental analysis calculated for $C_{38}H_{68}ErGeSi_2KN_2O_6$: C, 46.38; H, 6.96; N, 2.85. Found: C, 45.78; H, 6.91; N, 2.91.

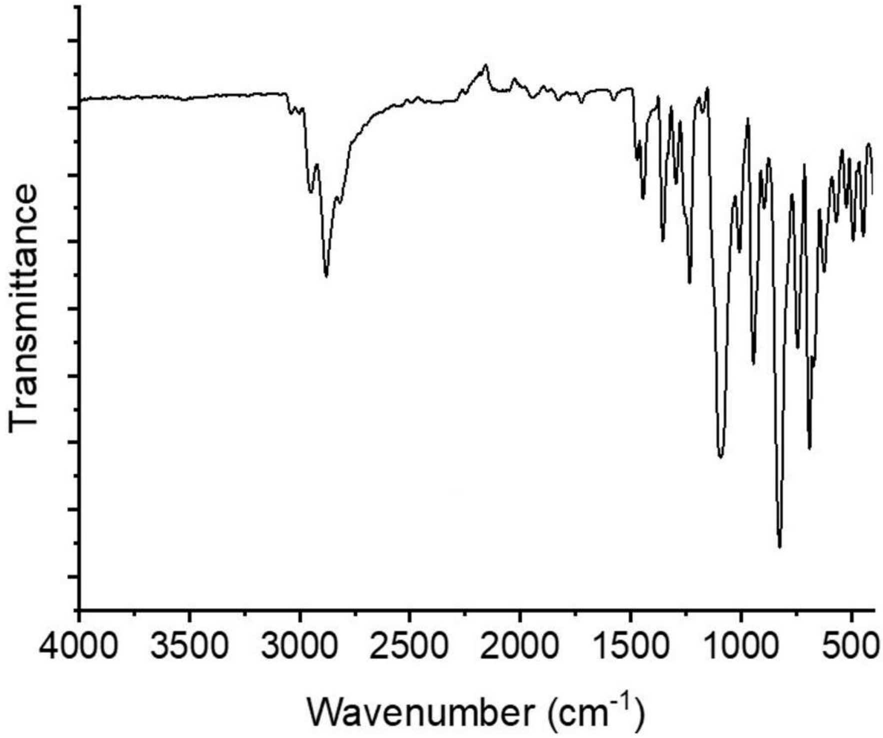

Figure S1. FTIR spectrum of solid [K(2.2.2-crypt)][$\mathbf{1}_{\mathbf{Dy}}$].

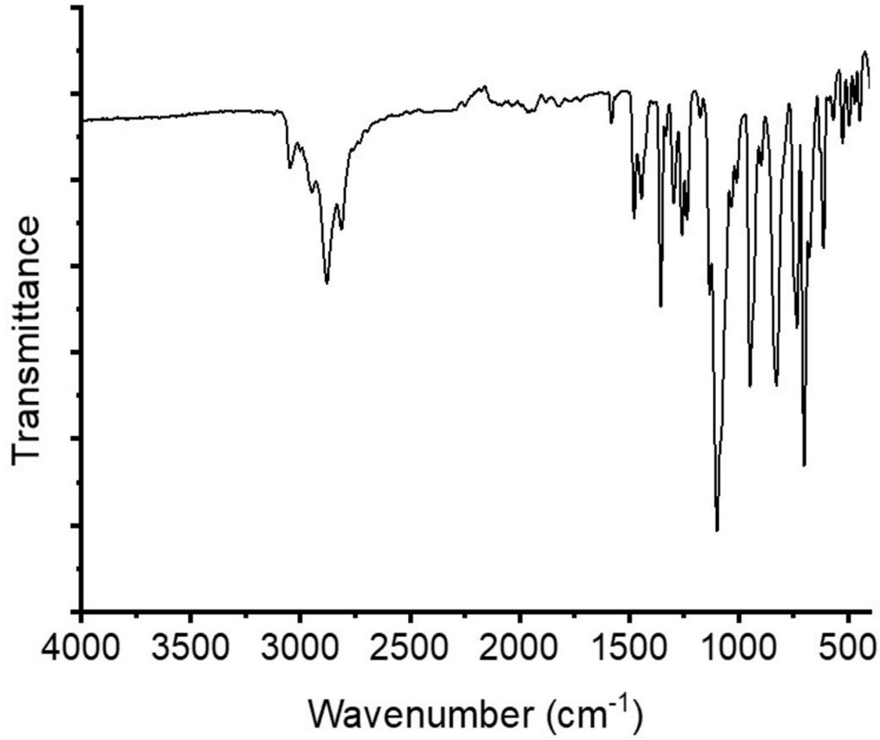

Figure S2. FTIR spectrum of solid [K(2.2.2-crypt)][$\mathbf{1}_{\mathbf{Er}}$].

X-Ray Crystallography

Single crystals of [K(2.2.2-crypt)][**1**$_{Dy}$] and [K(2.2.2-crypt)][**1**$_{Er}$] were covered with NVH oil (degassed and dried) and mounted on a Rigaku FR-007HF rotating anode equipped with a Saturn 724+ CCD area detector and a quarter-chi goniometer (dysprosium) or an Agilent XCalibur Gemini Ultra diffractometer (erbium). Cu-K$_{\alpha}$ radiation was used in both cases. Using Olex2,[17] structures were solved with the olex2.solve[18] structure solution program using charge flipping and refined with the SHELXL[19] refinement package using Least Squares minimization. Crystallographic data were deposited at Cambridge Crystallographic Data Centre. These data can be obtained free of charge from the Cambridge Crystallographic Data Centre via https://www.ccdc.cam.ac.uk/structures/.

Table S1. Crystal data and structure refinement for [K(2.2.2-crypt)][**1**$_{Dy}$] and [K(2.2.2-crypt)][**1**$_{Er}$].

	**[K(2.2.2-crypt)][1$_{Dy}$]**	**[K(2.2.2-crypt)][1$_{Er}$]**
CCDC Number	2223352	2223351
Empirical formula	$C_{38}H_{68}DyGeKN_2O_6Si_2$	$C_{38}H_{68}ErGeKN_2O_6Si_2$
Formula weight	979.31	984.07
Temperature/K	100	100
Crystal system	monoclinic	monoclinic
Space group	$P2_1/c$	$P2_1/c$
a/Å	13.1238(4)	13.09115(10)
b/Å	16.9156(4)	16.97080(9)
c/Å	20.2469(6)	20.19777(13)
α/°	90	90
β/°	102.449(3)	102.2514(6)
γ/°	90	90
V/Å^3	4389.1(2)	4385.09(5)
Z	4	4
ρ_{calc} /g cm^{-3}	1.482	1.491
μ/mm^{-1}	11.562	6.008
F(000)	2012.0	2020.0
Crystal size/mm^3	0.45 × 0.35 × 0.05	0.3 × 0.2 × 0.1
2θ range for data collection/°	6.878 to 136.12	8.656 to 144.24
Index ranges	$-15\leq h\leq15$, $-20\leq k\leq20$, $-23\leq l\leq24$	$-16\leq h\leq15$, $-20\leq k\leq20$, $-24\leq l\leq 24$
Reflections collected	22442	40137
Independent reflections	7815 [R_{int} = 0.0583, R_{sigma} = 0.0510]	8561 [R_{int} = 0.0406, R_{sigma} = 0.0213]
Data/restraints/parameters	7815/71/500	8561/0/469
Goodness-of-fit on F^2	1.128	1.066
Final R indexes [I>=2σ (I)]	R_1 = 0.0430, wR_2 = 0.1161	R_1 = 0.0283, wR_2 = 0.0771
Final R indexes [all data]	R_1 = 0.0487, wR_2 = 0.1261	R_1 = 0.0289, wR_2 = 0.0776
Largest diff. peak/hole / e Å^{-3}	1.18/-1.09	1.24/-0.89

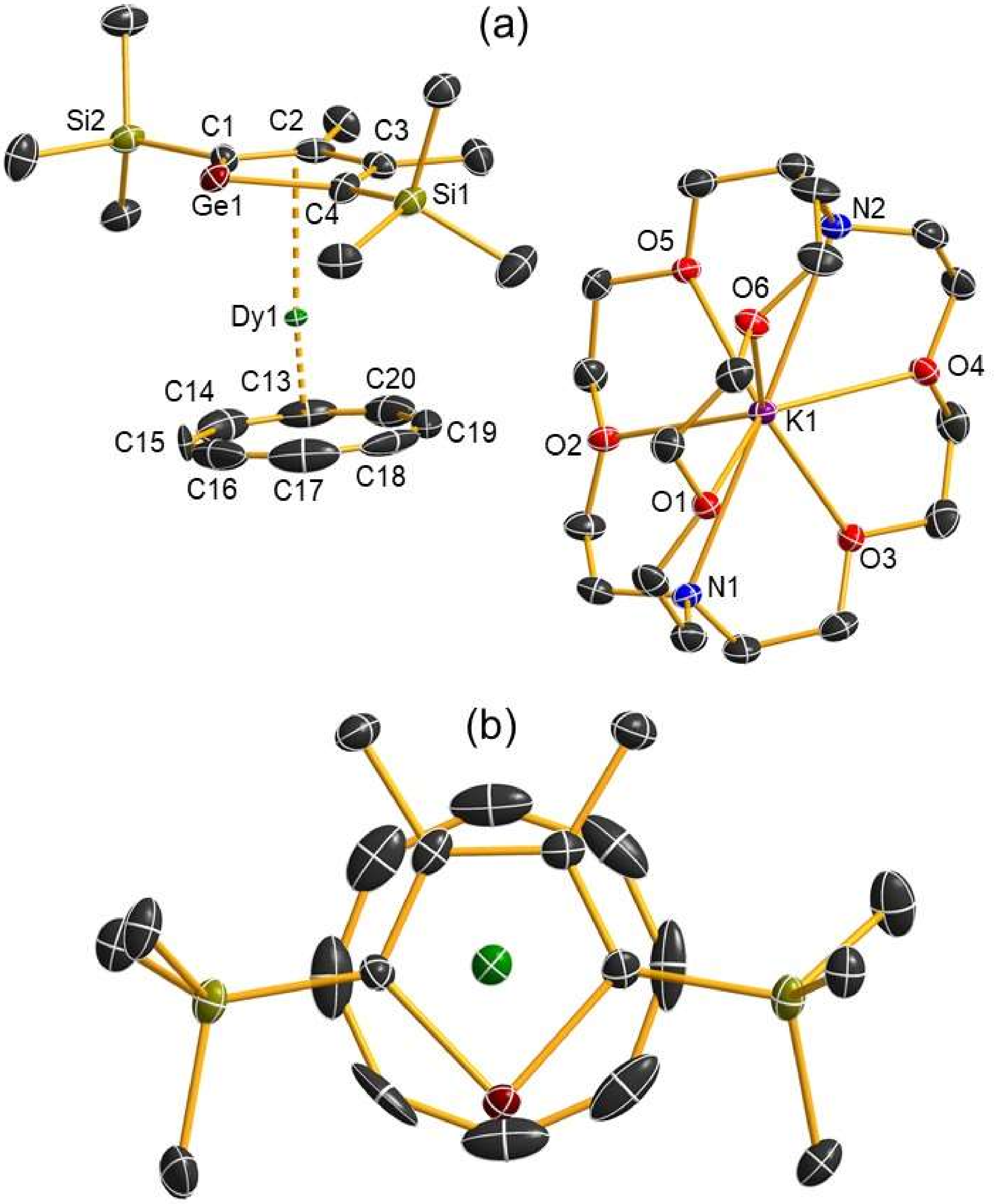

Figure S3. Thermal ellipsoid representation (50% probability) of the structure of [K(2.2.2-crypt)][$\mathbf{1}_{\mathbf{Dy}}$]. For clarity, hydrogen atoms are not shown.

Table S2. Selected distances and angles for $\mathbf{1}_{\mathbf{Dy}}$.

	**Length/Å**	**Angle/°**
Dy1-C1	2.637(4)	
Dy1-C2	2.623(4)	
Dy1-C3	2.632(4)	
Dy1-C4	2.620(4)	
Dy1-Ge1	2.9424(5)	
Dy1-C13	2.575(5)	
Dy1-C14	2.582(5)	
Dy1-C15	2.549(5)	
Dy1-C16	2.538(5)	
Dy1-C17	2.561(5)	
Dy1-C18	2.553(4)	
Dy1-C19	2.556(5)	
Dy1-C20	2.546(4)	
Ge1-C1	1.959(4)	
Ge1-C4	1.965(4)	
Dy-COT centroid	1.7956(2)	
Dy-Cp^{Ge} centroid	2.3063(2)	
COT-Dy-CpGe		175.135(12)

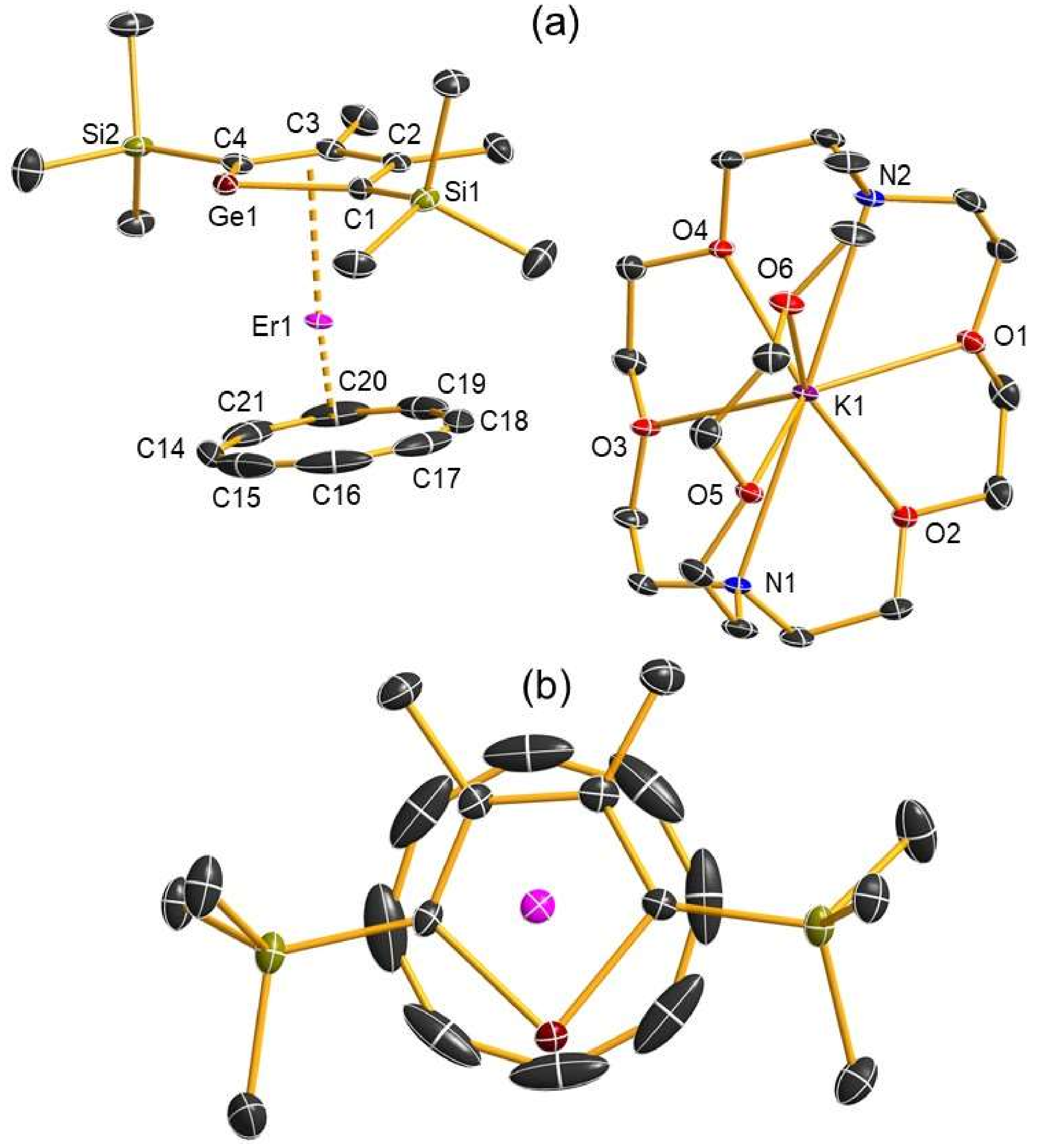

Figure S4. Thermal ellipsoid representation (50% probability) of the structure of [K(2.2.2-crypt)][$\mathbf{1}_{\mathbf{Er}}$]. For clarity, hydrogen atoms are not shown.

Table S3. Selected distances and angles for $\mathbf{1}_{\mathbf{Er}}$.

**Atom**	**Length/Å**	**Angle/°**
Er1-C1	2.589(2)	
Er1-C2	2.601(2)	
Er1-C3	2.596(2)	
Er1-C4	2.602(2)	
Er1-Ge1	2.9152(3)	
Er1-C14	2.545(3)	
Er1-C15	2.527(3)	
Er1-C16	2.533(3)	
Er1-C17	2.536(3)	
Er1-C18	2.534(3)	
Er1-C19	2.523(3)	
Er1-C20	2.541(3)	
Er1-C21	2.560(3)	
Ge1-C1	1.975(2)	
Ge1-C2	1.965(2)	
Er-COT centroid	1.7610(13) Å	
Er-Cp^{Ge} centroid	2.2693(10) Å	
COT-Er-Cp^{Ge}		175.38(5)°

Magnetic property measurements
Magnetic measurements were performed either using a Quantum Design MPMS3 SQUID magnetometer or a Quantum Design MPMS-XL7 SQUID magnetometer equipped with a 7 T magnet. Samples were prepared by gently crushing the crystalline materials before transferring them to a 7 mm NMR tube and covering them in eicosane. Then the tubes were flame sealed under a static vacuum. The eicosane was melted in a water bath at 40 °C to prevent crystallite torquing. Diamagnetic corrections were performed using Pascal's constants.[20]

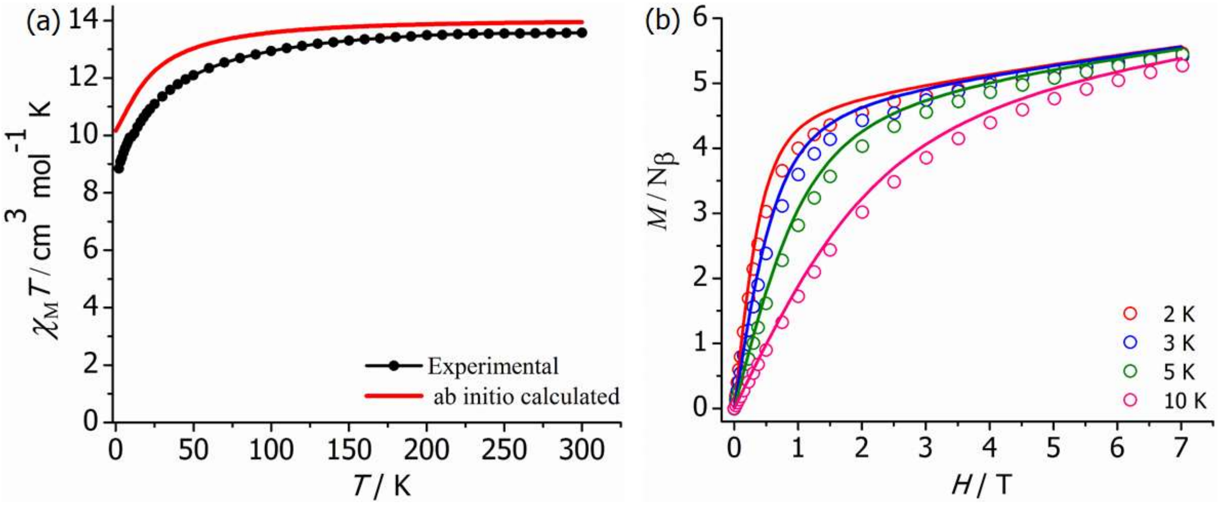

Figure S5. Experimental and ab initio calculated magnetic susceptibility (a) and isothermal field-dependent magnetization (b) plot for $\mathbf{1_{Dy}}$. The value of $\chi_M T$ at 300 K is 13.58 cm^3 K mol^{-1} and the value of M at 2.0 K and 7 T is 5.48 μ_B.

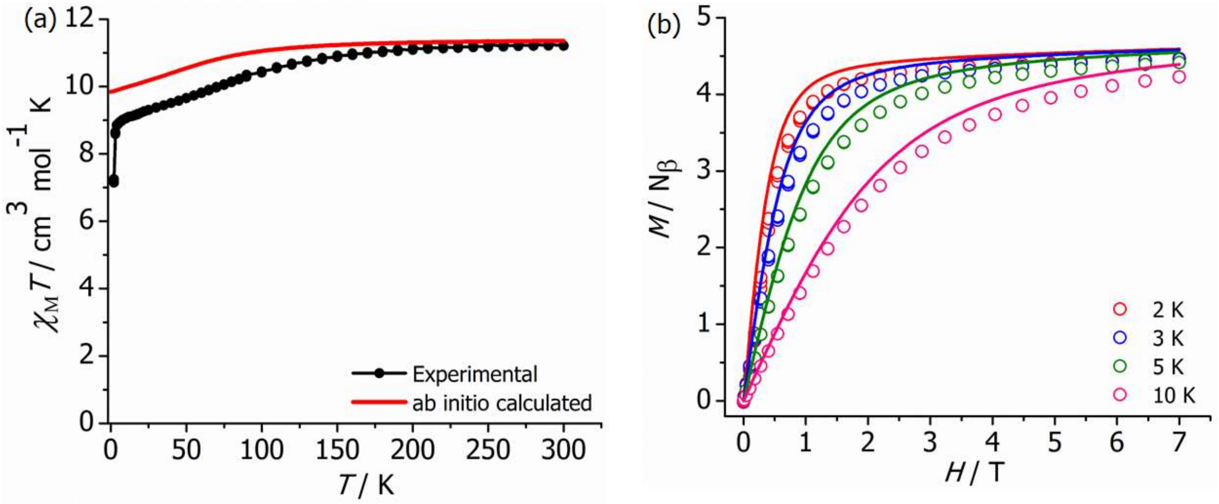

Figure S6. Experimental and ab initio calculated magnetic susceptibility (a) and isothermal field-dependent magnetization (b) plot for $\mathbf{1_{Er}}$. The value of $\chi_M T$ at 300 K is 11.17 cm^3 K mol^{-1} and the value of M at 2.0 K and 7 T is 4.47 μ_B.

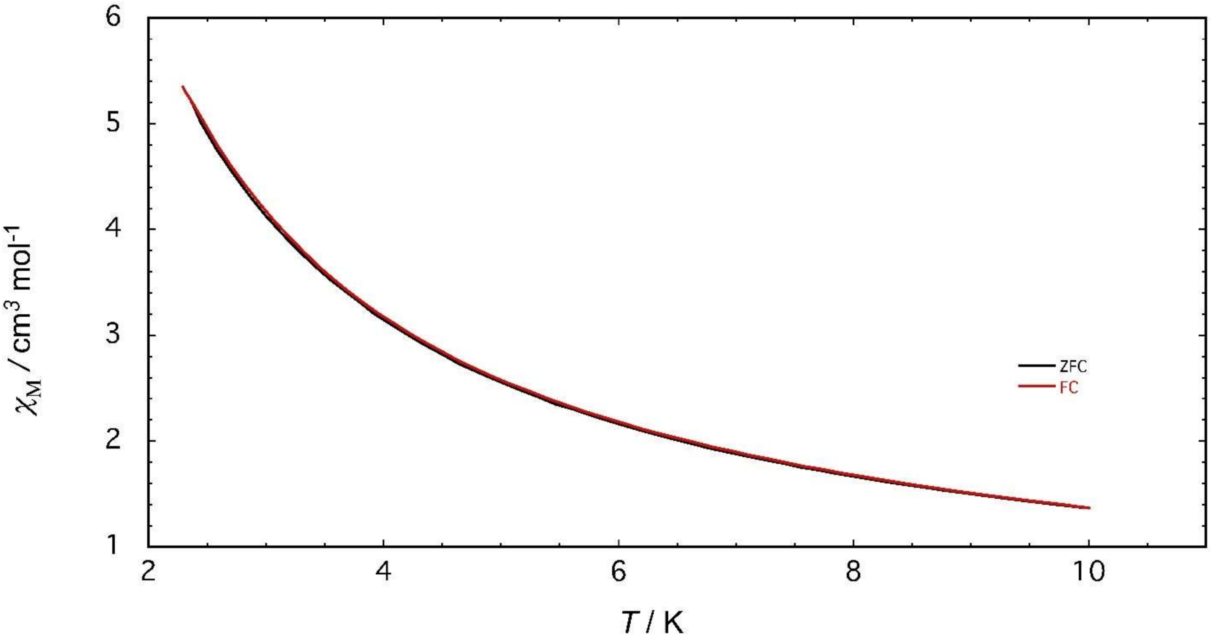

Figure S7. Plot of zero-field cooled (black line) and field-cooled (H_{DC} = 1400 Oe) magnetic susceptibility versus temperature for $\mathbf{1}_{\mathbf{Dy}}$.

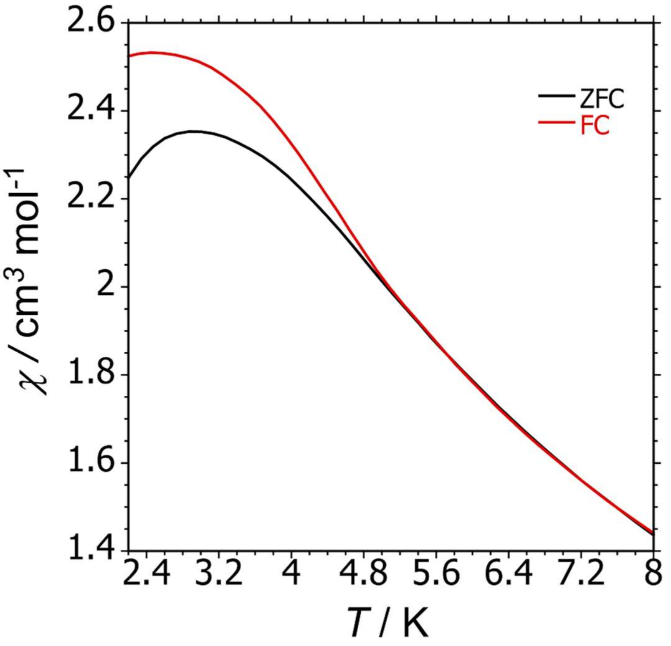

Figure S8. Plot of zero-field cooled (black line) and field-cooled (H_{DC} = 1400 Oe) magnetic susceptibility versus temperature for $\mathbf{1}_{\mathbf{Er}}$.

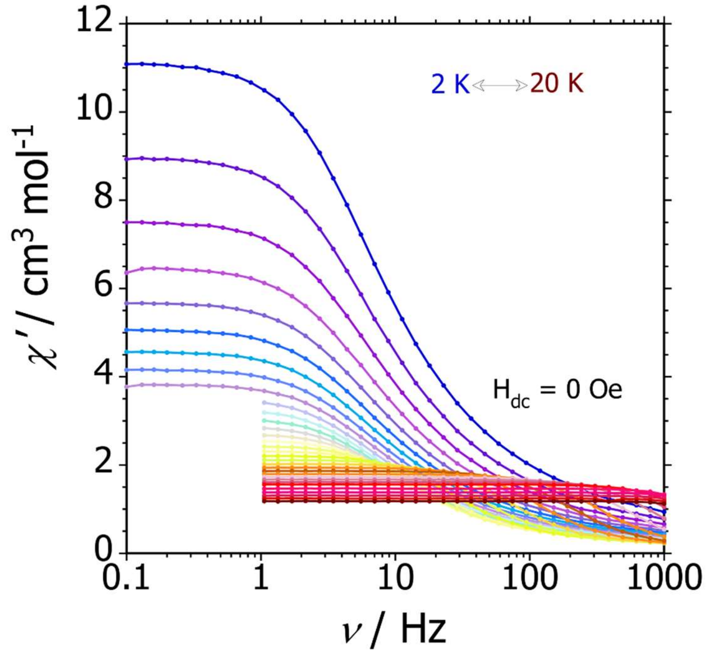

Figure S9. Frequency dependence of the in-phase susceptibility (χ') for $\mathbf{1}_{\mathbf{Er}}$ in zero DC field at ν = 1-1000 Hz and temperatures of 2-20 K.

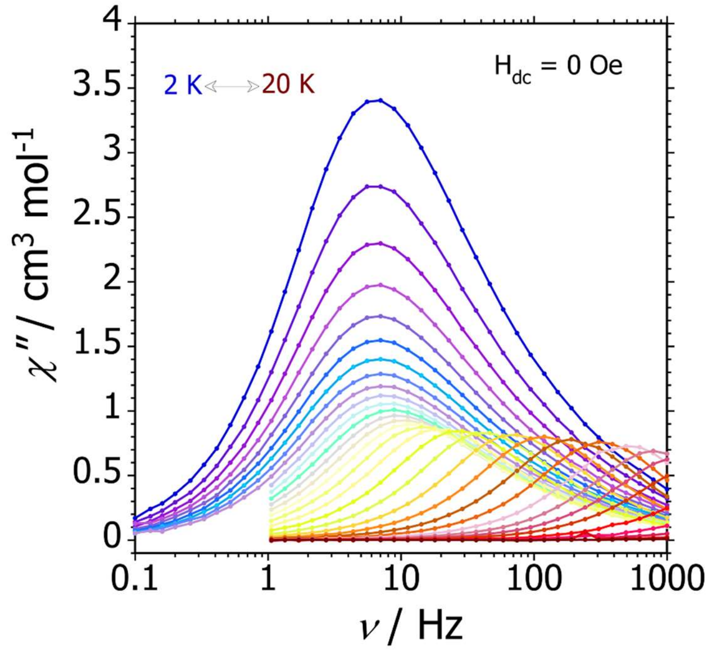

Figure S10. Frequency dependence of the out-of-phase susceptibility (χ'') for $\mathbf{1}_{\mathbf{Er}}$ in zero DC field at ν = 1-1000 Hz and temperatures of 2-20 K.

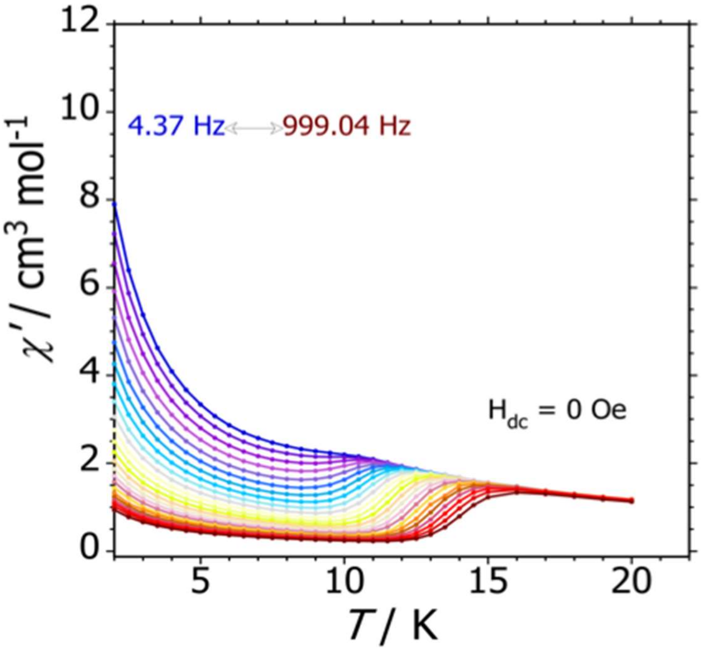

Figure S11. Temperature dependence of the in-phase susceptibility (χ') for $\mathbf{1}_{\mathbf{Er}}$ in zero DC field with ν = 4-999 Hz.

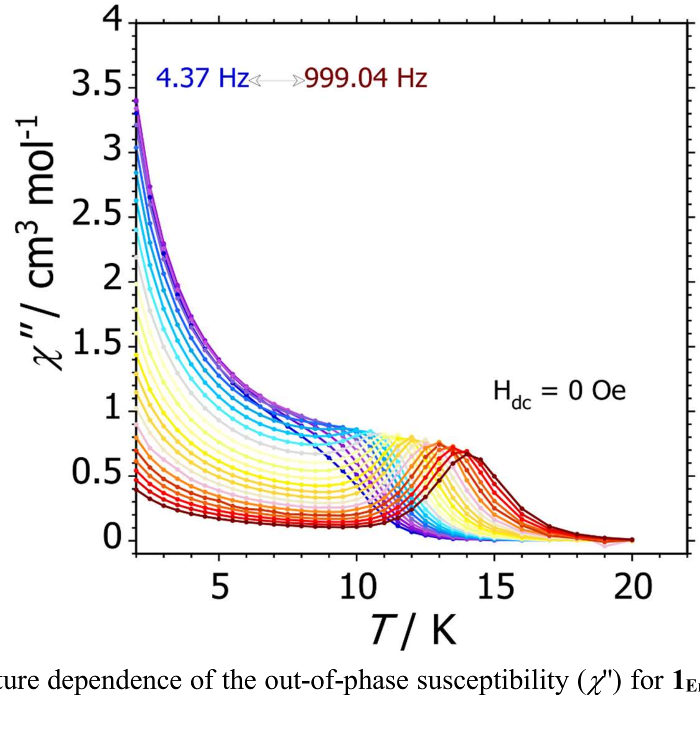

Figure S12. Temperature dependence of the out-of-phase susceptibility (χ'') for $\mathbf{1}_{\mathbf{Er}}$ in zero DC field with ν = 4-999 Hz.

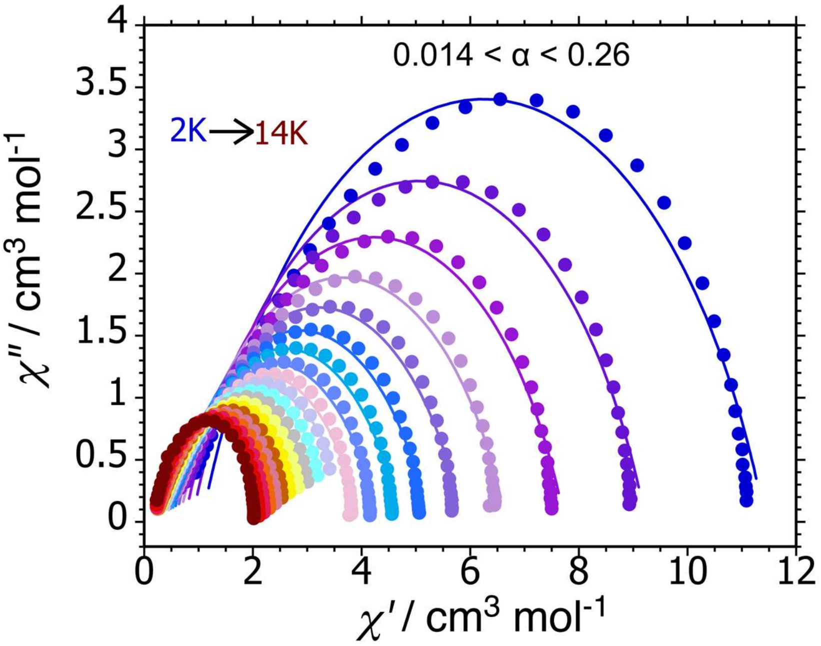

Figure S13. Cole-Cole plots for the AC susceptibilities in zero DC field for **1_{Er}** from 2-14 K. Solid lines represent fits to the data using equations 1 and 2.

$$\chi'(\nu_{ac}) = \chi_\infty + \frac{(\chi_s - \chi_\infty)[1 + (2\pi\nu_{ac}\tau)^{1-\alpha}\sin(\alpha\pi/2)]}{1 + 2(2\pi\nu_{ac}\tau)^{1-\alpha}\sin(\alpha\pi/2) + (2\pi\nu_{ac}\tau)^{2(1-\alpha)}} \quad \text{Equation 1}$$

$$\chi''(\nu_{ac}) = \frac{(\chi_s - \chi_\infty)(2\pi\nu_{ac}\tau)^{1-\alpha}\cos(\alpha\pi/2)}{1 + 2(2\pi\nu_{ac}\tau)^{1-\alpha}\sin(\alpha\pi/2) + (2\pi\nu_{ac}\tau)^{2(1-\alpha)}} \quad \text{Equation 2}$$

Table S4. Relaxation fitting parameters for $\mathbf{1}_{\mathbf{Er}}$ corresponding to Figure S13.

T/K	χ_T/cm^{3}mol^{-1}	χ_S/cm^{3}mol^{-1}	α	τ/s
1.99991	11.43195	1.06202	0.26025	0.01892
2.49938	9.23401	0.8751	0.26015	0.01874
2.99938	7.73741	0.74089	0.26148	0.01854
3.49926	6.65456	0.6442	0.26213	0.01833
4.0001	5.84973	0.57302	0.26237	0.01816
4.49991	5.21643	0.51713	0.26086	0.01792
4.99989	4.70299	0.47363	0.25761	0.01758
5.50007	4.28499	0.43862	0.25258	0.01719
6.00001	3.9296	0.41192	0.24416	0.01664
6.49987	3.80494	0.34794	0.27883	0.01749
7.00017	3.52522	0.3331	0.26507	0.01645
7.49986	3.2759	0.3212	0.24826	0.01526
7.99986	3.05754	0.30975	0.23052	0.01399
8.50001	2.85833	0.29917	0.21086	0.01258
9.00017	2.68436	0.28868	0.18979	0.01105
9.49984	2.5252	0.27803	0.16518	0.00926
9.99992	2.37999	0.26541	0.13975	0.00724
10.49992	2.25188	0.25306	0.11058	0.0052
10.99969	2.1385	0.23802	0.08652	0.00344
11.49985	2.03619	0.22062	0.06599	0.00214
12.00001	1.94711	0.214	0.0497	0.00131
12.49994	1.86708	0.21067	0.03753	7.91E-04
12.99989	1.79325	0.21641	0.02212	4.87E-04
13.49982	1.72817	0.23151	0.01449	3.12E-04
14.00011	1.66654	0.28535	8.18E-11	2.04E-04

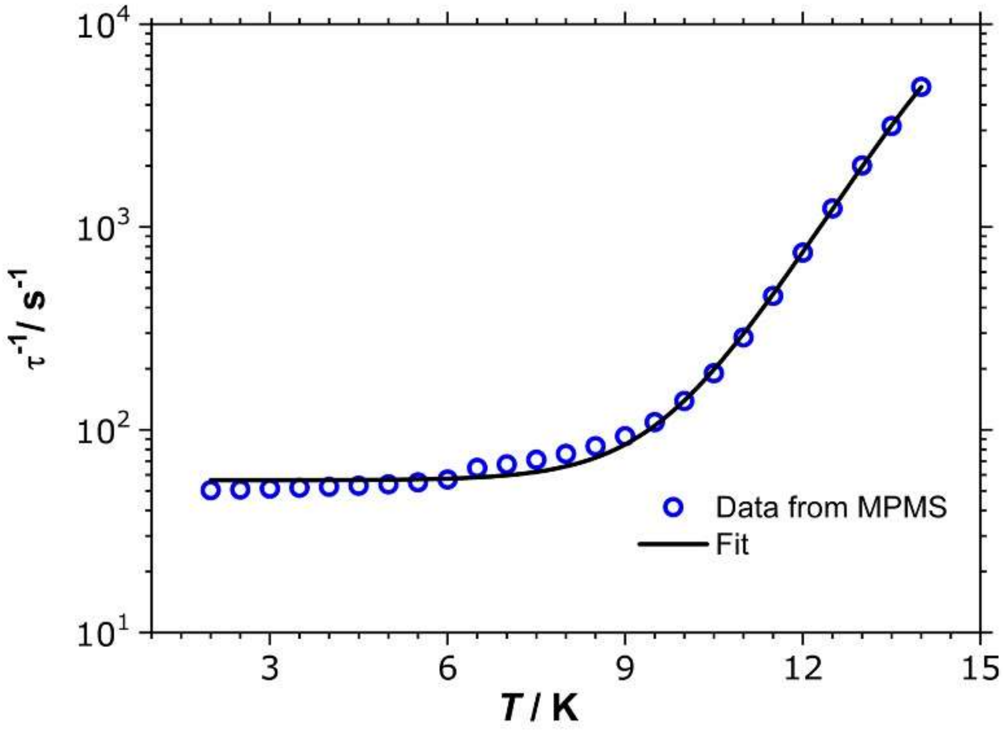

Figure S14. Plot of τ^{-1} *vs.* temperature for $\mathbf{1}_{\mathbf{Er}}$ (H_{DC} = 0 Oe). The blue points are from the AC susceptibility measurements. The solid black line is the best fit (adjusted R^2 = 0.99992) to $\tau^{-1} = \tau_0^{-1} e^{-U_{\mathrm{eff}}/k_{\mathrm{B}}T} + CT^n + \tau_{QTM}^{-1}$, giving: U_{eff} = 120(1) cm^{-1}, $\tau_0 = 10^{-9.02(4)}$ s, $C = 10^{-1.6(4)}$ s^{-1} K^{-n}, n = 3.4(4) and $\tau_{\mathrm{QTM}} = 10^{-1.7(1)}$ s.

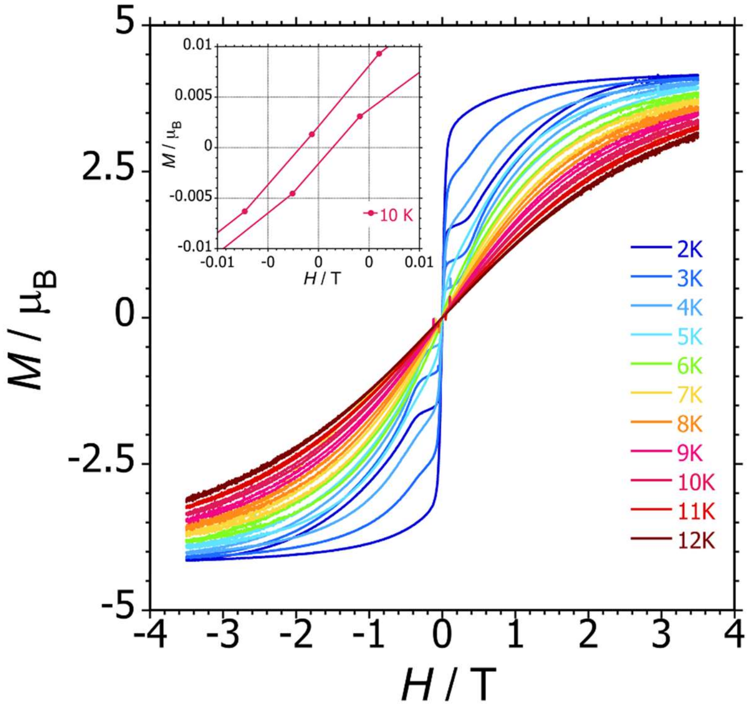

Figure S15. Magnetic hysteresis loops for $\mathbf{1_{Er}}$ with an average field-sweeping rate of 200 Oe s^{-1}.

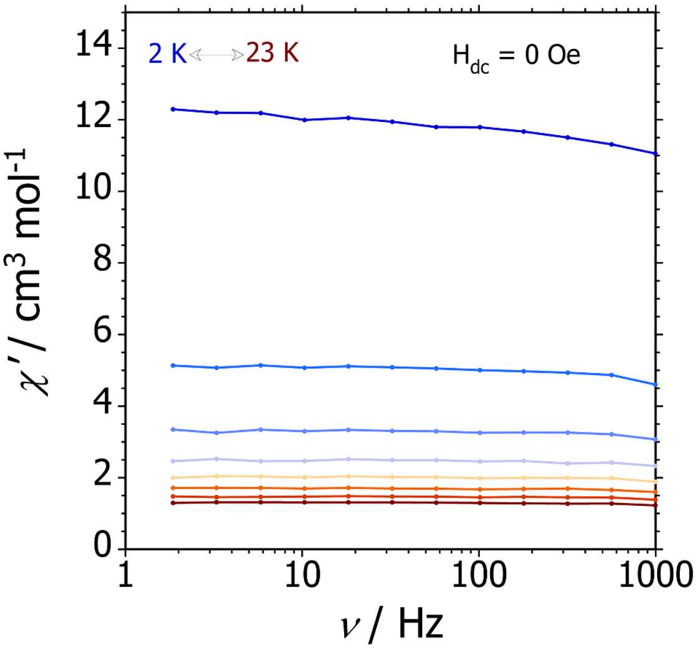

Figure S16. Frequency dependence of the in-phase susceptibility (χ') for $\mathbf{1_{Dy}}$ in zero DC field at ν= 1-1000 Hz and temperatures of 2-20 K.

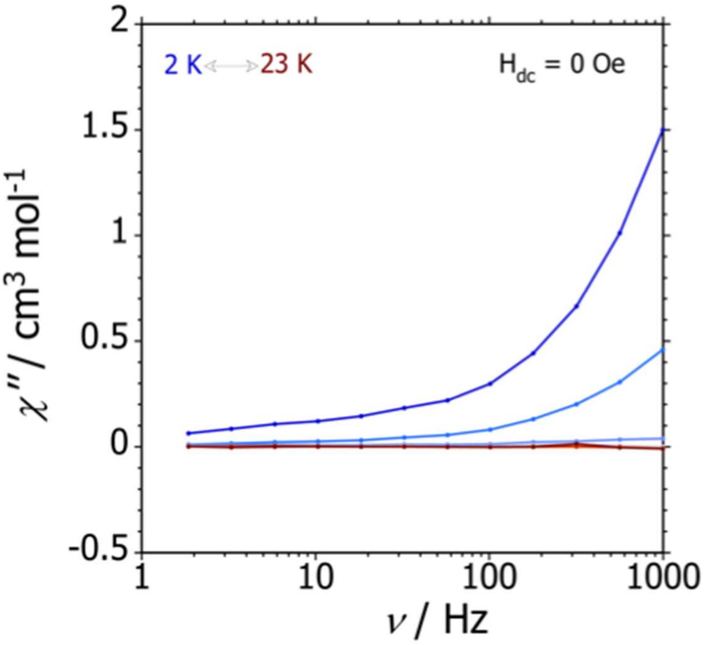

Figure S17. Frequency dependence of the out-of-phase susceptibility (χ'') for $\mathbf{1_{Dy}}$ in zero DC field at ν = 1-1000 Hz and temperatures of 2-20 K.

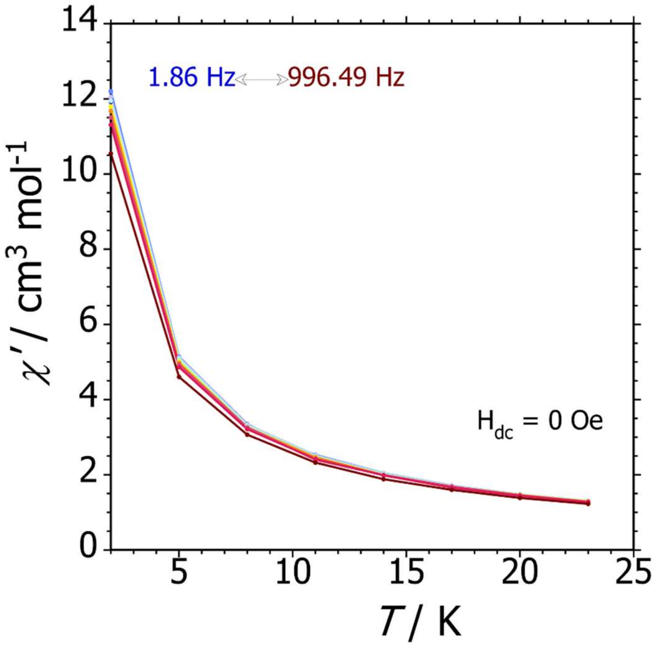

Figure S18. Temperature dependence of the in-phase susceptibility (χ') for $\mathbf{1_{Dy}}$ in zero DC field with ν = 4-999 Hz.

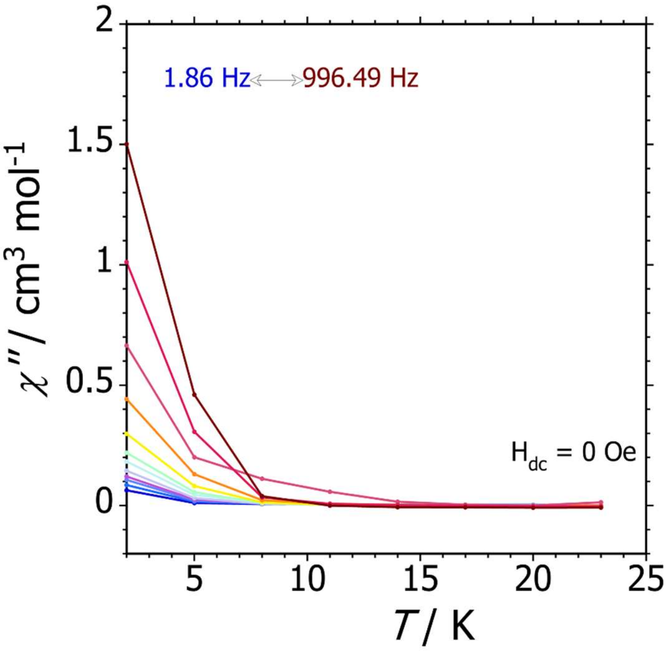

Figure S19. Temperature dependence of the out-of-phase susceptibility (χ'') for $\mathbf{1_{Dy}}$ in zero DC field with ν = 4-999 Hz.

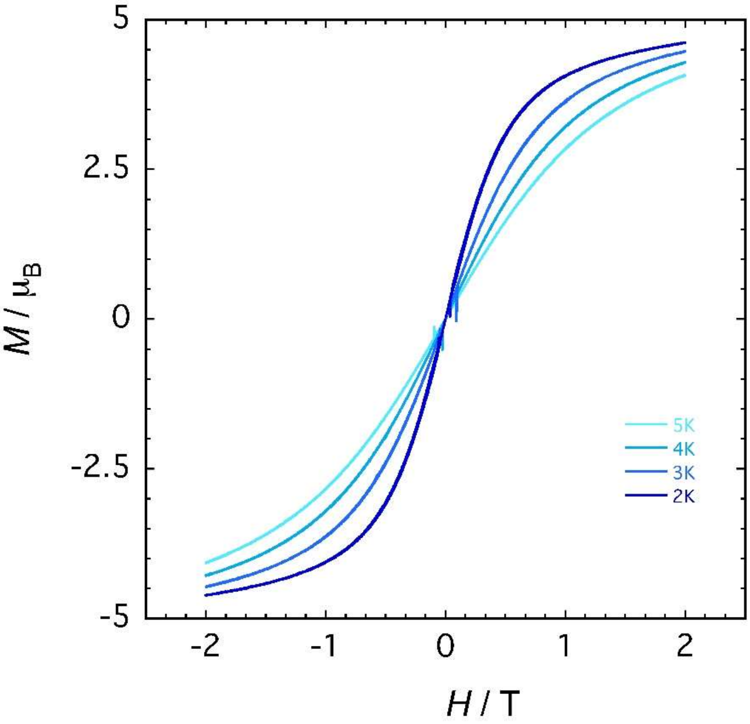

Figure S20. Magnetic hysteresis for $\mathbf{1_{Dy}}$ with an average field-sweeping rate of 20 mT s^{-1}.

Computational details
All calculations were carried using the coordinates for non-hydrogen atoms obtained from the X-ray structure using the ORCA 5.0.2 software package.[21] Hydrogen atom positions were optimized at the DFT level using the pure GGA PBE exchange correlation functional,[22,23] with the positions of other atoms fixed. The calculations were of the CASSCF/QDPT/Single_Aniso type, and the DKH (Douglas-Kroll-Hess) Hamiltonian was used throughout the calculations to account for relativistic effects. We employed the SARC2-DKH-QZVP basis set for the lanthanide ions, whereas all other atoms were treated with the DKH-def2-TZVP basis set in combination with 'AutoAux' auxiliary basis set.[24-26] In the active space, we considered 11 electrons in 7 f-orbital CAS (7,11) for Er^{3+} and 9 electron in 7 f-orbital CAS (7,9) for Dy^{3+}. Furthermore, 35 quartet and 90 doublet states for Er^{3+} and 21 sextet, 128 quartet and 130 doublet states for Dy^{3+} were solved in the state-averaged (SA) calculations. To consider the spin-orbit coupling, we used the quasi-degenerate perturbation theory (QDPT) approach using SA-CASSCF wave functions.[27] The Single_Aniso module[28] as implemented in ORCA was used to compute the *g*-tensor and crystal-field parameters of the low-lying excited state using previously calculated spin-orbit states.

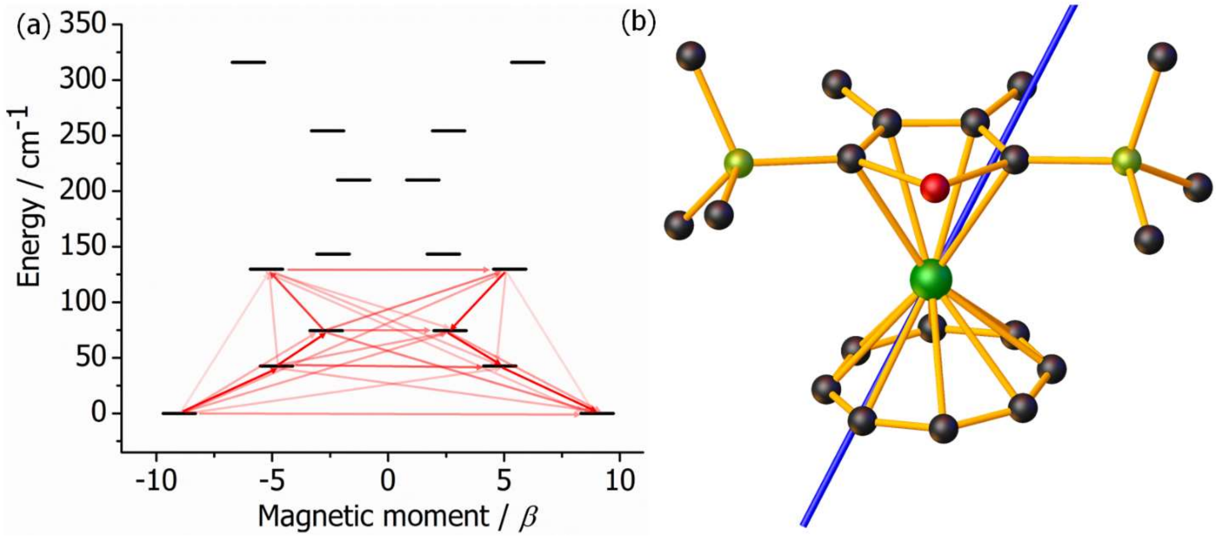

Figure S21. (a) Calculated relaxation barrier for $\mathbf{1_{Dy}}$. Stronger red arrows indicate larger absolute value of the transition magnetic moment matrix elements between the respective states. Transitions involving higher-energy states not involved in the relaxation mechanism are omitted for clarity. (b) Orientation of the anisotropy axes (blue arrow) of the ground Kramer's doublet for $\mathbf{1_{Dy}}$.

Table S5. Calculated energy (cm^{-1}) of the low-lying spin-orbit states for $\mathbf{1_{Er}}$ and $\mathbf{1_{Dy}}$.

$\mathbf{1_{Er}}$	$\mathbf{1_{Dy}}$
0.000	0.000
0.000	0.000
102.6102	42.6817
102.6102	42.6817
118.5969	74.4865
118.5969	74.4865
137.9732	129.9045
137.9732	129.9045
151.8361	143.3998
151.8361	143.3998
181.3979	210.1103
181.3979	210.1103
204.4805	254.3708
204.4805	254.3708
246.8812	316.0064
246.8812	316.0064

Table S6. Computed energy of the KDs, g-tensors and wavefunction compositions for **1**$_{\mathbf{Er}}$.

<table>
<tr><th>KDs</th><th>E / (cm^{-1})</th><th>g_x</th><th>g_y</th><th>g_z</th><th>Wavefunction composition</th></tr>
<tr><td>1</td><td>0.000</td><td>0.003</td><td>0.004</td><td>17.74</td><td>97.09%|±15/2>+0.70%|±11/2>+1.89%|±9/2></td></tr>
<tr><td>2</td><td>102.610</td><td>11.61</td><td>6.92</td><td>1.154</td><td>59.45%|±1/2>+29.61%|±3/2>+8.07%|±5/2>+1.84%|±7/2>+0.36%|±9/2></td></tr>
<tr><td>3</td><td>118.597</td><td>1.528</td><td>3.904</td><td>6.764</td><td>25.39%|±1/2>+45.96%|±3/2>+20.60%|±5/2>+5.41%|±7/2>+1.19%|±9/2>0.70%|±11/2></td></tr>
<tr><td>4</td><td>137.973</td><td>1.742</td><td>3.188</td><td>11.64</td><td>6.28%|±1/2>+11.28%|±3/2>+31.62%|±5/2>+22.80%|±7/2>+3.20%|±9/2>+4.65%|±11/2>+19.80%|±13/2></td></tr>
<tr><td>5</td><td>151.836</td><td>1.129</td><td>2.220</td><td>11.380</td><td>4.73%|±1/2>+10.65%|±3/2>+38.33%|±5/2>+25.27%|±7/2>+5.78%|±9/2>+2.20%|±11/2>+12.81%|±13/2>+0.20%|±15/2></td></tr>
<tr><td>6</td><td>181.39</td><td>0.035</td><td>0.581</td><td>15.11</td><td>3.17%|±1/2>+1.38%|±3/2>+0.85%|±5/2>+25.52%|±7/2>+27.01%|±9/2>+23.42%|±11/2>+17.75%|±13/2>+0.86%|±15/2></td></tr>
<tr><td>7</td><td>204.481</td><td>0.386</td><td>0.619</td><td>12.63</td><td>0.57%|±1/2>+0.89%|±3/2>+0.16%|±5/2>+19.01%|±7/2>+37.53%|±9/2>+3.93%|±11/2>+36.98%|±13/2>+0.86%|±15/2></td></tr>
<tr><td>8</td><td>246.881</td><td>0.086</td><td>0.174</td><td>15.74</td><td>0.37%|±1/2>+0.18%|±3/2>+0.18%|±5/2>+0.04%|±7/2>+22.97%|±9/2>+64.23%|±11/2>+11.57%|±13/2>+0.43%|±15/2></td></tr>
</table>

Table S7. Computed energy of the KDs, g-tensors and wavefunction compositions for $\mathbf{1_{Dy}}$.

KDs	E / (cm^{-1})	g_x	g_y	g_z	Wavefunction composition
1	0.000	0.0411	0.067	18.02	73.56%|±15/2>+3.80%|±13/2>+15.16%|±11/2>+5.11%|±7/2>+1.26%|±5/2>
2	42.682	0.2650	0.3197	13.80	3.26%|±15/2>+26.15%|±13/2>+1.88%|±11/2>+24.82%|±9/2>+4.68%|±7/2>+8.59%|±5/2>+15.89%|±3/2>+14.68%|±1/2>
3	74.487	0.0221	0.5607	10.28	4.0%|±15/2>+18.92%|±13/2>+4.23%|±11/2>+6.28%|±9/2>+7.96%|±7/2>+13.28%|±5/2>+13.30%|±3/2>+31.97%|±1/2>
4	129.905	0.1280	0.2828	15.34	15.73%|±15/2>+8.64%|±13/2>+22.12%|±11/2>+0.59%|±9/2>+21.13%|±7/2>+15.69%|±5/2>+8.32%|±3/2>+7.73%|±1/2>
5	143.400	0.8925	1.4747	8.50	0.06%|±15/2>+21.49%|±13/2>+6.48%|±11/2>+2.94%|±9/2>+8.56%|±7/2>+14.72%|±5/2>+35.13%|±3/2>+10.53%|±1/2>
6	210.110	4.5669	3.5299	1.5562	0.57%|±15/2>+6.0%|±13/2>+16.02%|±11/2>+18.28%|±9/2>+14.93%|±7/2>+27.87%|±5/2>+2.44%|±3/2>+13.83%|±1/2>
7	254.371	13.385	7.50	1.190	0.13%|±15/2>+1.86%|±13/2>+9.20%|±11/2>+20.63%|±9/2>+18.68%|±7/2>+9.03%|±5/2>+21.22%|±3/2>+19.21%|±1/2>
8	316.006	0.000	0.000	19.86	2.64%|±15/2>+13.08%|±13/2>+24.85%|±11/2>+26.39%|±9/2>+18.89%|±7/2>+9.50%|±5/2>+3.42%|±3/2>+1.18%|±1/2>

Table S8. Calculated transition magnetic moment matrix elements (in Bohr magneton) for $\mathbf{1}_{\mathbf{Er}}$.

Climbing Transition			Crossing Transition		
Initial KD	Final KD	Magnitude	Initial KD	Final KD	Magnitude
1	2	0.26264	1	1	0.00133
1	3	0.92235	1	2	0.07505
1	4	1.04527	1	3	0.20538
1	5	0.76435	1	4	0.09679
1	6	0.55533	1	5	0.12977
1	7	0.88027	1	6	0.05446
1	8	0.62196	1	7	0.01834
2	3	2.65999	1	8	0.00195
2	4	0.63235	2	2	2.24165
2	5	0.457858	2	3	1.66513
2	6	0.184817	2	4	0.77594
2	7	0.295056	2	5	0.64116
2	8	0.26450	2	6	0.20870
3	4	1.89753	2	7	0.21250
3	5	1.59115	2	8	0.10283
3	6	0.82450	3	3	1.37364
3	7	0.76227	3	4	1.12308
3	8	0.24154	3	5	0.90687
4	5	1.91315	3	6	0.18487
4	6	0.92034	3	7	0.32191
4	7	1.59035	3	8	0.17678
4	8	0.69577	4	4	1.06534
5	6	2.06225	4	5	0.80681
5	7	1.20515	4	6	0.61875
5	8	0.59598	4	7	0.38087
6	7	1.41423	4	8	0.08708
6	8	0.74872	5	5	0.69202
7	8	2.31672	5	6	0.39737
			5	7	0.55483
			5	8	0.10562
			6	6	0.11630
			6	7	0.32802
			6	8	0.14119
			7	7	0.17765
			7	8	0.11230
			8	8	0.04787

Table S9. Calculated transition magnetic moment matrix elements (in Bohr magneton) for $\mathbf{1_{Dy}}$.

Climbing Transition			Crossing Transition		
Initial KD	Final KD	Magnitude	Initial KD	Final KD	Magnitude
1	2	1.97512	1	1	0.01803
1	3	1.85784	1	2	0.04900
1	4	0.72085	1	3	0.04225
1	5	0.31230	1	4	0.01597
1	6	0.10149	1	5	0.04424
1	7	0.08381	1	6	0.04492
1	8	0.09672	1	7	0.01240
2	3	2.92236	1	8	0.00081
2	4	2.31781	2	2	0.12419
2	5	1.05697	2	3	0.13691
2	6	0.17462	2	4	0.03018
2	7	0.05081	2	5	0.09497
2	8	0.10601	2	6	0.06686
3	4	1.87691	2	7	0.09750
3	5	3.44223	2	8	0.00368
3	6	0.27973	3	3	0.13954
3	7	0.15502	3	4	0.06900
3	8	0.27338	3	5	0.20395
4	5	1.23998	3	6	0.20124
4	6	1.43899	3	7	0.12637
4	7	0.07927	3	8	0.00436
4	8	1.85009	4	4	0.08138
5	6	3.69567	4	5	0.18303
5	7	0.19234	4	6	0.36531
5	8	0.79750	4	7	0.19849
6	7	3.20424	4	8	0.00739
6	8	0.10139	5	5	0.51660
7	8	0.06938	5	6	0.8828
			5	7	0.37572
			5	8	0.63018
			6	6	1.13341
			6	7	2.40807
			6	8	0.02666
			7	7	2.75976
			7	8	0.05917
			8	8	0.00022

Table S10. Computed crystal field parameters for $\mathbf{1_{Er}}$ and $\mathbf{1_{Dy}}$.

k	*q*	$\mathbf{1_{Er}}$	$\mathbf{1_{Dy}}$
	-2	-0.5965E-01	-0.1455E+00
	-1	-0.2852E+00	0.1082E+00
2	0	-0.2803E+00	-0.5360E+00
	1	0.6281E+00	-0.9227E+00
	2	-0.9847E-01	0.5297E+00
	-4	-0.5805E-03	-0.5824E-02
	-3	0.8034E-02	0.6700E-01
	-2	-0.1824E-02	-0.1999E-01
	-1	-0.3619E-02	0.3038E-02
4	0	-0.5640E-02	-0.3569E-02
	1	0.5989E-03	-0.5351E-02
	2	-0.2221E-02	0.2033E-01
	3	0.5457E-02	-0.4510E-01
	4	-0.1041E-03	0.4911E-02
	-6	0.5134E-05	-0.4524E-04
	-5	0.3080E-03	0.3642E-04
	-4	-0.2475E-04	0.6607E-04
	-3	0.2104E-03	-0.1794E-03
	-2	-0.1141E-04	0.2533E-04
	-1	0.6587E-04	0.2926E-04
6	0	-0.1750E-04	0.3365E-05
	1	-0.1426E-03	-0.5285E-05
	2	-0.4651E-04	-0.7435E-04
	3	0.1547E-03	0.5421E-04
	4	-0.2610E-06	-0.2123E-04
	5	0.4016E-01	0.1430E-03
	6	0.8376E-01	-0.3087E-04

References

[15] Z. Dong, C. R. W. Reinhold, M. Schmidtmann, T. Müller, *Organometallics* **2018,** *37*, 4736.
[16] S. M. Cendrowski-Guillaume, M. Nierlich, M. Lance, M. Ephritikhine, *Organometallics* **1998,** *17*, 786.
[17] O. V. Dolomanov, L. J. Bourhis, R. J. Gildea, J. A. K. Howard, H. Puschmann, *J. Appl. Cryst.* **2009,** *42*, 339.
[18] L. J. Bourhis, O. V. Dolomanov, R. J. Gildea, J. A. K. Howard, H. Puschmann, *Acta Cryst. Section A* **2015,** *71*, 59.
[19] G. Sheldrick, *Acta Cryst. Section C* **2015,** *71*, 3.
[20] G. A. Bain, J. F. Berry, *J. Chem. Ed.* **2008,** *85*, 532.
[21] F. Neese, *WIREs Comp. Mol. Sci.* **2022,** *12*, e1606.
[22] J. P. Perdew, K. Burke, M. Ernzerhof, *Phys. Rev. Lett.* **1997,** *78*, 1396.
[23] J. P. Perdew, K. Burke, M. Ernzerhof, *Phys. Rev. Lett.* **1996,** *77*, 3865.
[24] D. Aravena, F. Neese, D. A. Pantazis, *J. Chem. Theory Comput.* **2016,** *12*, 1148.
[25] J. Chmela, M. E. Harding, *Mol. Phys.* **2018,** *116*, 1523.
[26] F. Weigend, R. Ahlrichs, *PCCP* **2005,** *7*, 3297.
[27] D. Ganyushin, F. Neese, *J. Chem. Phys.* **2006,** *125*, 024103.
[28] L. F. Chibotaru, L. Ungur, *J. Chem. Phys.* **2012,** *137*, 064112.
